# Analysis of Primary Metabolites in Cabbage (*Brassica* *oleracea* var. *capitata*) Varieties Correlated with Antioxidant Activity and Taste Attributes by Metabolic Profiling

**DOI:** 10.3390/molecules24234282

**Published:** 2019-11-25

**Authors:** Ryota Mabuchi, Mao Tanaka, Chihori Nakanishi, Nanako Takatani, Shota Tanimoto

**Affiliations:** Faculty of Human Culture and Science, Prefectural University of Hiroshima, 1-1-71, Ujina-Higashi, Minami-ku, Hiroshima 734-8558, Japans-tanimoto@pu-hiroshima.ac.jp (S.T.)

**Keywords:** cabbage, metabolomics, GC-MS, DPPH, ORAC, electronic tongue, OPLS

## Abstract

*Brassica* vegetables, such as cabbage, have many health benefits arising from their antioxidant and anticancer properties. These properties are endowed by the metabolite composition of the plant, and it is therefore important to elucidate the metabolic profile and associated activities in this genus. This study objectively evaluated the characteristics of cabbage varieties using metabolic profiling to identify the primary metabolic components that correlate with antioxidant activity and taste attributes. GC-MS analysis was used to identify the primary metabolites. Antioxidant activity was measured by oxygen radical absorbance capacity (ORAC) and 2,2-diphenyl-1-picrylhydrazyl radical (DPPH) scavenging assays, and an electronic tongue was used to quantitate nine taste attributes. Orthogonal projections to latent structures (OPLS) using SIMCA 14 correlated the metabolite components with the taste and antioxidant characteristics. We identified 4-aminobutyric acid, fructose 1-phosphate, adipic acid, 5-oxoproline, *N*-acetylglycine, *O*-phosphoethanolamine, and homovanillic acid as important determinants of DPPH scavenging activity and umami, sourness, acidic bitterness, irritant and saltiness, bitterness, astringency, and richness, respectively. These metabolites represent markers indicating breed differences and contribute to differential cabbage functionality. These studies could be extended to measure additional metabolites, as well as to understand the role of growth conditions on the metabolic profile and health benefits of plants.

## 1. Introduction

*Brassica* vegetables, including cabbage, broccoli, and cauliflower, are widely eaten around the world as both fresh produce and processed products. These vegetables contain unique compounds that provide them with distinctive tastes, nutritional values, and health applications. The flavor of *Brassica* vegetables has been attributed to isothiocyanates generated from the hydrolysis of glucosinolates [1,2]. Both the glucosinolates and isothiocyanates are vital nutrients that have been associated with the prevention of cancer [2,3]. These vegetables are also reported to have a high concentration of antioxidant compounds, such as phenols and flavonoids [4,5]. Owing to the clear benefits provided by their consumption, a complete library of the range of components in this plant genus would be of great use for a variety of biological and biochemical industries.

Food metabolomics, a key technique used in foodomics, is the best way to identify characteristic metabolic components among distinct crop varieties [6,7,8,9]. Multiple chemical components of *Brassica* have already been characterized through metabolomics [9,10,11,12,13,14,15,16]. Kim et al. were able to distinguish Chinese and Korean cabbages using proton nuclear magnetic resonance (^1^H-NMR) spectroscopy [10]. Jeon et al. differentiated the primary and secondary metabolic components of green and purple pak choi using gas chromatography-time-of-flight mass spectrometry (GC-TOF-MS) and high- performance liquid chromatography-ultraviolet (HPLC-UV) detection [11]. However, a correlation between these characteristic components and the taste and antioxidant properties of the vegetables has not yet been determined.

In-depth studies of cabbage (*Brassica oleracea*) would be highly informative, as a wide variety of subspecies has been created worldwide through extensive breeding and crossbreeding. For example, Japan has multiple cabbage varieties grown in different places, during distinct seasons, and for specific uses (raw, cooked, and processed). These varieties display unique appearances and disease resistance and thus can also be expected to differ in their taste and metabolic compositions. However, an accurate characterization of the components in these cabbage varieties is far from complete.

This study used metabolic profiling to characterize the antioxidant activity and taste attributes of six Japanese cabbage varieties. Water-soluble primary metabolic components were identified by GC-MS analysis. The antioxidant activity and taste attributes were measured using biochemical assays and an electronic tongue, respectively. Multivariate analysis of these data identified 4-aminobutyric acid, fructose 1-phosphate, adipic acid, 5-oxoproline, *N*-acetylglycine, *O*-phosphoethanolamine, and homovanillic acid as potential breed-specific components underlying the antioxidant activity and taste of each variety.

## 2. Results and Discussion

### 2.1. Annotated Water-Soluble Primary Metabolites by GC-MS Analysis

Six varieties of Japanese cabbage were evaluated in this study: YR-ginjiro (Y), Satou-kun (S), Okina (O), Kinkei-201go (KI), Kogetsu-SP (KO), and Hiro-kanran (H). GC-MS was used to obtain the total ion chromatograms (TICs) for each variety (Figure S1). Annotation of the TIC peaks revealed the presence of 331 unique metabolites (Table S1). The number of annotated metabolites by cabbage variety was: 301 (Y), 278 (S), 284 (O), 281 (KI), 287 (KO), and 274 (H). The majority of these primary metabolites were amino acids, organic acids, and carbohydrates. Although some of these compounds resembled primary metabolites found during earlier metabolomics studies of *Brassica* [11,17,18], these previous studies only identified about 40–50 primary metabolic components [11,18]. Previous studies had used GC-OF-MS for qualitative and quantitative metabolic profiling; however, the peaks in our work were detected using the retention index, and the metabolic components were comprehensively annotated based on their match rate with the commercially available GC/MS Metabolite Component Database (Ver. 2, Shimadzu). This metabolic fingerprinting method allowed for the identification of a far greater number of metabolic components, a desirable feature when attempting to correlate a chemical profile with the complex activities and attributes of an organism.

### 2.2. In Vitro Antioxidant Assay of DPPH and ORAC

**Figure 1:**
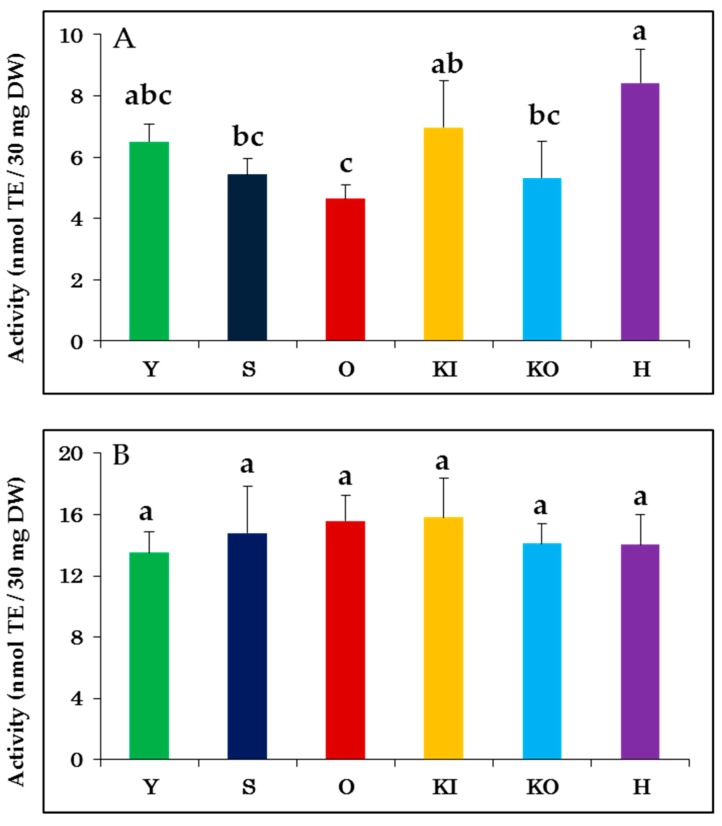
ANOVA test of antioxidant activity measured by (**A**) DPPH scavenging and (**B**) oxygen radical absorbance capacity (ORAC). Y: YR-Ginjiro, S: Satou-kun, O: Okina, KI: Kinkei-201go, KO: Kogetsu-SP, H: Hiro-kanran. TE: Trolox equivalent; DW: Dry weight of sample. The values are mean ± SD (*n* = 5). Significant differences (*p* < 0.05) between the cabbage varieties are indicated by different letters. The *p*-values between each group are shown in Table S2.

Established methods exist for measuring the antioxidant activity of food and other agricultural products. These methods generally use either hydrogen atom transfer (HAT) or single electron transfer (SET) for detecting antioxidant activity, and it is ideal to use both techniques to ensure an accurate characterization [19,20]. In our study, the antioxidant activity was measured using oxygen radical absorbance capacity (ORAC) and 2,2-diphenyl-1-picrylhydrazyl radical (DPPH) scavenging activity as the HAT- and SET-based techniques, respectively (Table S1). The average antioxidant activity of the six cabbage varieties measured by DPPH scavenging and ORAC was 6.21 ± 1.37 and 14.6 ± 0.92 activity, respectively, which are similar to previously reported values [18,21]. A one-way analysis of variance (ANOVA) test was used to compare the antioxidant activities of the cabbage varieties (Figure 1). While there was no significant difference observed between the varieties using ORAC (*p* = 0.463, Figure 1B), the antioxidant activity of the different breeds varied significantly during DPPH scavenging (*p* < 0.05, Figure 1A). These differences were further confirmed using Tukey’s multiple comparison procedure. From these analyses, it could be concluded that cabbage variety H showed the highest antioxidant activity during DPPH scavenging, while the lowest activity was observed for O. The different antioxidant activities observed for the cabbage varieties within and between the two tests are reflective of the different antioxidant mechanisms measured by the assays [21,22]. As such, it appears that the six cabbage varieties have similar antioxidants or antioxidant mechanisms with respect to ORAC, but unique compounds or pathways for DPPH scavenging.

### 2.3. Measurement of Taste Attributes by Electronic Tongue

The traditional method for evaluating the taste of foods, the sensory test, involves an experienced evaluator (called a sensory panelist) tasting the sample. However, this method suffers from low objectivity and reproducibility and requires substantial labor from the panelists and their trainers. The electronic tongue functions as an alternative to this process; it can be used to more objectively identify and quantify tastes [23] and has already been successfully applied to a variety of foods [23,24,25]. There are a small, but growing, number of reported applications of the electronic tongue to *Brassica* vegetables, including a recent study on the taste evaluation of broccoli flowers [26].

**Figure 2:**
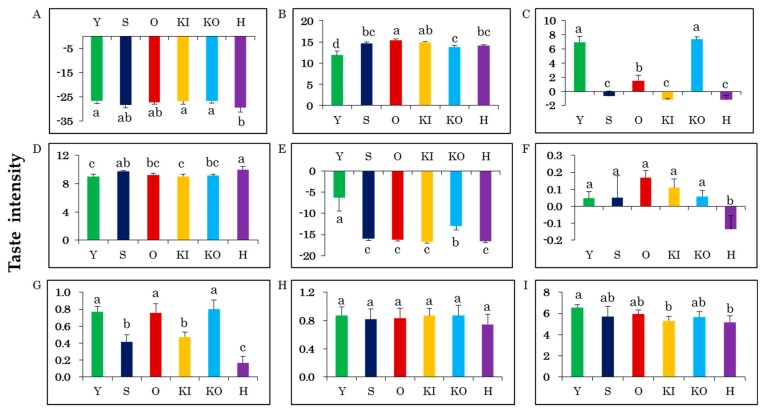
Taste attribute measurements of cabbage varieties using an electronic tongue. (**A**) Sourness, (**B**) acidic bitterness, (**C**) irritant, (**D**) umami, (**E**) saltiness, (**F**) bitterness, (**G**) astringency, (**H**) richness, (**I**) sweetness. The values are mean ± SD (*n* = 5). Significant differences (*p* < 0.05) between the cabbage varieties are indicated by different letters. The *p*-values between each group are shown in Table S2.

We used the electronic tongue to quantify the different tastes among the cabbage varieties (Table S1). Of the nine taste attributes measured, differences among the varieties were detected for all tastes except richness (Figure 2). H had the lowest sourness value and was significantly different from KI, KO, and Y, which had the highest value (Figure 2A). Y, KO, and O were significantly different in acidic bitterness, with O having the highest and Y the lowest acidic bitterness value (Figure 2B). Most of the varieties were distinct in irritant measurements, with Y and KO showing the highest values, while H, S, and KI showed negative values for this attribute (Figure 2C). H showed the highest umami value and was significantly different from the other varieties, except for S (Figure 2D). Saltiness distinguished Y and KO from each other and from the other four varieties (Figure 2E). H was the least bitter and astringent variety, differing significantly from all other varieties in both of these tastes (Figure 2F,G). Astringency also varied significantly between groups of “Y, O, KO” and “S, KI” (Figure 2G). H and KI were the least sweet cabbage varieties, while Y was significantly sweeter than all other varieties (Figure 2I). These results show the utility of the electronic tongue to quantitatively evaluate the tastes of cabbage varieties.

### 2.4. Metabolic Profiling of Varieties by Principal Component Analysis (PCA) and OPLS-Discriminant Analysis (DA)

**Figure 3:**
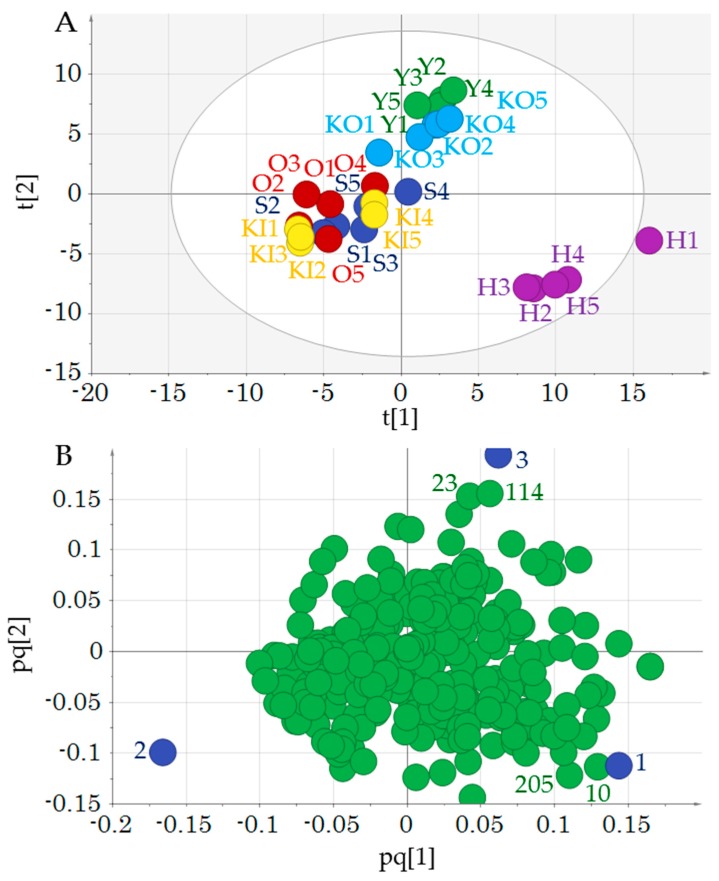
Orthogonal projections to latent structures-discriminant analysis **(**OPLS-DA) of cabbage variety metabolites. (**A**) Score plots and (**B**) loading plots for the three cabbage groups identified during the analysis (group 1: H; group 2: KI, O, and S; group 3: KO and Y). Letters and numbers in (**A**) indicate sample ID and numbers in (**B**) indicate group identity (blue circle) (Table S1). Numbers adjacent to green circles correspond to *x* variable IDs in Table S1: Alanine (10), 3-hydroxyisobutyric acid (23), 5-oxoproline (114), and *O*-phosphoethanolamine (205). Cross-validated (CV)-ANOVA was used to calculate the *p*-value of this model (*p* = 0.00000046).

A data set was created consisting of the metabolic components as *x* variables, and the antioxidant and taste values as *y* variables. This data set was subjected to statistical analysis using SIMCA 14 (MKS Instruments). We initially performed PCA-X analysis on the metabolites to gain an overview of the cabbage varieties (Figure S2). However, the relationship between the metabolites and the cabbage varieties was unclear. Therefore, an OPLS-DA analysis was performed for better discrimination. Numbers 1–6 were assigned to each variety and a discriminant analysis was performed in OPLS-DA (Figure S3). This was autofitted with a 3 + 1 + 0 model and provided an R2Y = 0.524 and Q2 = 0.365, indicating that the model was not significant (*p* = 0.91). H was separated from the other varieties in component 1 (t[1]), while component 2 (t[2]) clustered KI, O, and S in the positive direction and Y and KO in the negative direction (Figure 3A). Varieties that showed similar trends were grouped and reanalyzed by OPLS-DA (group 1: H, group 2: KI, O, and S, group 3: KO and Y). This model was autofitted as 2 + 1 + 0 with R2Y = 0.867 and Q2 = 0.798, indicating a significant model (*p* < 0.05). This model gave an R2Y intercept = 0.584 and a Q2 intercept = −0.343 in a model accuracy by permutation test (*n* = 200). While the R2Y intercept was somewhat high, the Q2 intercept was less than 0, confirming some degree of model accuracy. In particular, H was distinct from the other varieties, likely reflecting that it is a wild species long cultivated in Japan, whereas the other varieties have been crossbred. These genetic differences are likely expressed through the metabolic profile. On the other hand, groups 2 (KI, O, and S) and 3 (KO and Y) differ in their cultivation areas, which have been shown to affect metabolites in cabbage plants [10]. This is likely due to differences in soil nutrients present in the cultivation fields.

The metabolites contributing to these varietal differences are shown in the loading plot (Figure 3B). The components that correlated with H were *O*-phosphoethanolamine and alanine. *O*-phosphoethanolamine is a metabolite involved in the synthesis of phosphatidylcholine, the main phospholipid of eukaryotic cells in plants [27]. Alanine is an amino acid that exhibits sweetness and umami. Studies evaluating free amino acids among inbred varieties of cabbage have shown varietal differences in alanine content [28]. Group 3 (KO and Y) was correlated with 3-hydroxyisobutyric acid and 5-oxoproline. The compound 3-hydroxyisobutyric acid is a metabolic intermediate of valine, and 5-oxoproline is a derivative amino acid of glutamine. Both are metabolites involved in amino acid biosynthesis. Metabolites may be associated with physiological differences between plant varieties; however, it is difficult to ascribe a specific function to these metabolite differences. Nevertheless, metabolites can serve as characteristic markers of cabbage varieties.

**Figure 4:**
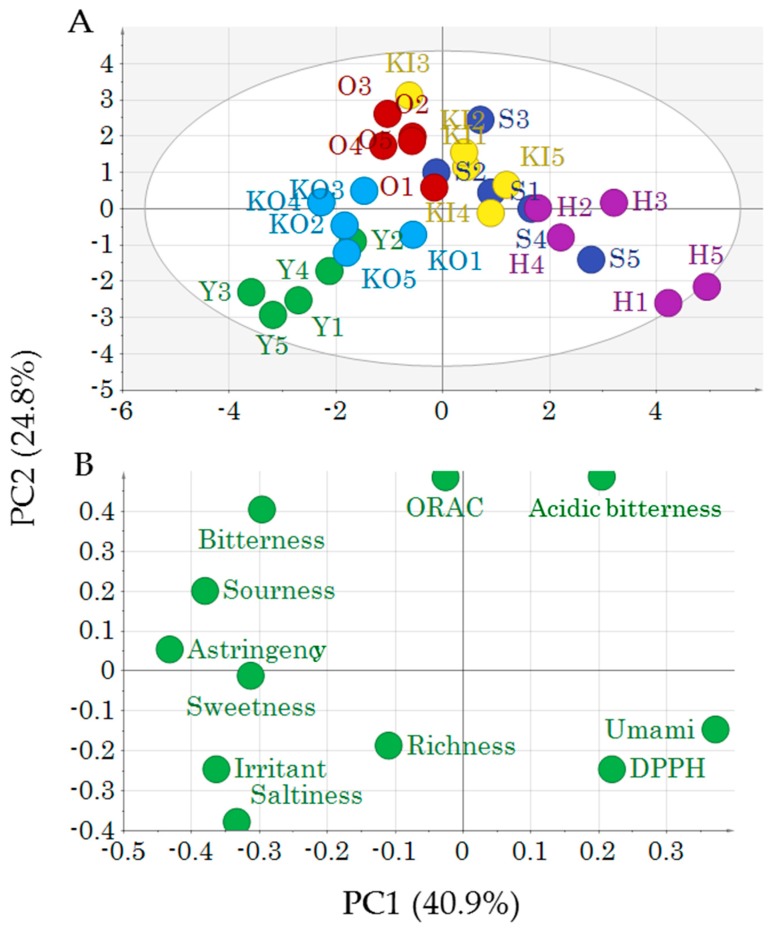
(**A**) Score plots and (**B**) loading plots of principal component analysis (PCA)-Y results for the different cabbage varieties. Letters and numbers in (**A**) indicate sample ID (Table S1).

PCA-Y was then performed to reveal the relationship between the *y* variables and the varieties. In the score plots (Figure 4A), the first principal component separates H in the positive direction from Y and KO in the negative direction. KI and O cluster together in the second principal component in the positive direction separately from H and Y in the negative direction. H is correlated with umami and DPPH scavenging, and Y is correlated with irritant and saltiness taste attributes (Figure 4B). Differences in the cabbage variety and cultivation area were reflected in taste attributes and antioxidant properties, as was observed with the metabolic profile. For example, KO and Y are cultivated in the same soil and cluster together in our analysis, indicating that the soil used for cultivation likely affects the taste and antioxidant properties of cabbage. This association will require further verification.

In *Brassica* plants, such as cabbage, the compounds responsible for bitter and irritant taste attributes are isothiocyanates and glucosinolate [29], while ascorbic acid and phenol components are known to provide antioxidant activity targeting DPPH [30,31,32]. These are often characterized as secondary metabolic components of plants. In our work, variety Y has a strong irritant taste and would therefore be expected to contain more isothiocyanates and glucosinolate than other varieties. Likewise, H, which has a high antioxidant activity against DPPH, should have higher antioxidant content. However, these secondary metabolic components were not investigated in this study but will provide avenues for future analyses.

### 2.5. OPLS Correlation Analysis of Metabolic Profiling with Antioxidant Activity and Taste Attributes 

**Figure 5:**
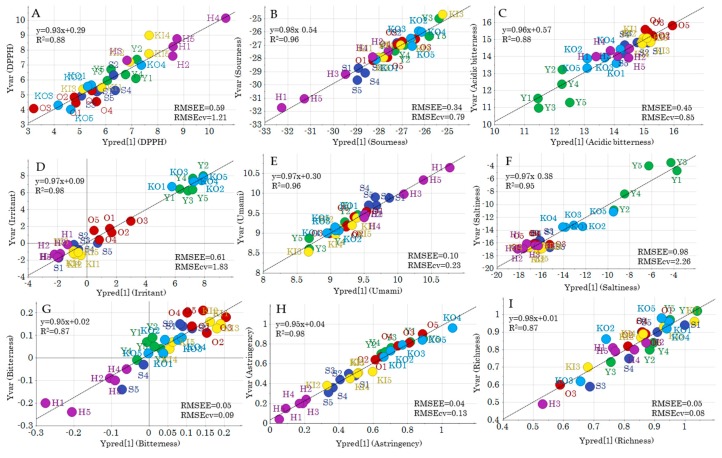
Regression analysis of the OPLS predictive model for each *y* variable. (**A**) DPPH, (**B**) sourness, (**C**) acidic bitterness, (**D**) irritant, (**E**) umami, (**F**) saltiness, (**G**) bitterness, (**H**) astringency, (**I**) richness. Alphanumeric labels indicate the sample IDs (Table S1). RMSEE: Root mean square errors of estimation; RMSEcv: Root mean square errors of cross-validation.

OPLS analysis was performed for each *y* variable to identify the metabolites correlated with these specific characteristics (Table 1). Statistically significant models could be created for all *y* variables, except ORAC and sweetness. The relationship between the variables and the different cabbage varieties could be visualized by regression analysis of each *y* variable and the predicted value from the corresponding model (Figure 5). From these analyses, H could be positively correlated with DPPH activity and umami and negatively correlated to sourness, astringency, and bitterness. Y shows a positive correlation with saltiness and a negative correlation with acidic bitterness. Irritant was positively correlated only with KO and Y. Although a significant predictive model could be created for richness, no differences between the varieties were observed. 

Values for the *x* variables important for prediction (VIP) were calculated for the *y* variables for which it was possible to create significant models and observe varietal differences. A VIP value of 1.0 or higher is considered to indicate an important *x* variable for the model [33], which varies between cabbage varieties. Many metabolites showed VIP values of 1.0 or more (Table S3). The metabolites showing the highest VIP values for their respective *y* variables are: 4-aminobutyric acid (DPPH and umami), fructose 1-phosphate (sourness), adipic acid (acidic bitterness), 5-oxoproline (irritant and saltiness), *N*-acetylglycine (bitterness), *O*-phosphoethanolamine (astringency), and homovanillic acid (richness). These primary metabolic components are considered candidate markers for each breed that provide the basis for its characteristic taste and antioxidant activity.

In this metabolomic study, electronic tongue and in vitro antioxidant assays were used to evaluate the differences in primary metabolites between cabbage varieties. These primary metabolites were correlated to the taste attributes and antioxidant activity of the cabbages. Previous studies in microalgae, *Prunus dulcis* leaves, and *Neptunia oleracea* [34,35,36] evaluated the correlation between antioxidant activity and metabolites based on metabolomics alone. Japanese sake, coffee, and fish have been analyzed using the electronic tongue, as well as metabolomic measurements [37,38,39,40]. This study successfully extends these methods into cabbage varieties to correlate their metabolite content with their characteristic taste and antioxidant activities. This approach could be extended to additional cabbage varieties and for evaluating the specific characteristics of individual cabbage varieties.

## 3. Materials and Methods

### 3.1. Chemicals

All reagents were obtained from commercial sources and were of guaranteed reagent. Methanol, chloroform, pyridine, and ribitol for GC-MS analysis were purchased from Wako Pure Chemicals Industries, Ltd. (Osaka, Japan). Methoxyamine hydrochloride and *N*-methyl-*N*-(trimethylsilyl)trifluoroacetamide (MSTFA) for GC derivatization were purchased from Sigma-Aldrich (St. Louis, MO, USA) and GL Sciences (Tokyo, Japan), respectively. DPPH and 2-morpholinoethanesulfonic acid (MES) for measuring DPPH activity were purchased from Sigma-Aldrich and TCI (Tokyo, Japan), respectively. Fluorescein and 2,2′-azobis(2-amidinopropane) dihydrochloride for use in the ORAC assay were purchased from Sigma-Aldrich and Wako, respectively. Trolox (Sigma-Aldrich) was used as a standard for DPPH and ORAC assays.

### 3.2. Experimental Samples

Six varieties of cabbage (H, O, KI, S, KO, and Y) cultivated in Kure City, Hiroshima Prefecture, Japan were used for this study. All varieties were harvested in December 2017, when they reached approximately equal maturity. H, O, KI, and S were cultivated in the same field, and KO and Y were cultivated in other fields in the same region. H has long been cultivated in Hiro-Machi, Kure City, Hiroshima Prefecture. As H has not been crossbred with other varieties, it closely resembles the wild variety, native species cultivated in Japan from an earlier period. KO is bright green, harvested in winter, and has resistance towards diseases such as yellow dwarf disease. KI is an extremely fast-growing variety, and the head shows excellent hypertrophy. O is one of the representative cabbage varieties in Japan. It is resistant to yellow dwarf disease and relatively fast growing. The head is shaped like an oblate spheroid and has excellent shape stability. S has slightly larger outer leaves, a slightly flat head containing yellow leaves, and no anthocyanin. Y has dark green leaves and can be harvested in winter. Five heads of each variety were used for this work. The samples were prepared as follows: Approximately two outer leaves were peeled from the head, the remaining parts were cut into halves, and the inner core portion of the head was removed. The sample was then thinly chopped with a cabbage slicer (RCS-70, Happy Japan, Yamagata, Japan), put in a bag with a chuck, and stored at −80 °C prior to analysis.

### 3.3. GC-MS Analysis

Details for GC-MS sample preparation and analysis have been described in our previous reports [39,40,41]. A portion of the sample stored at −80 °C was lyophilized (FDU-1200, EYELA, Tokyo, Japan) overnight and then ground to a powder using a mill (TUBE-MILLC S001, IKA, Staufen, Germany) for 30 s at 25,000 rpm. Mixed solutions of methanol/ultrapure water/chloroform (2.5/1/1 *v*/*v*/*v*, 1 mL) and ribitol (internal standard, 0.2 mg/mL, 60 µL) were added to 50 mg of the powdered sample. After stirring for 5 min, the mixture was centrifuged (16000× *g*, 0 °C, 5 min). Ultrapure water (400 µL) was added to 800 µL of the supernatant (methanol phase), which was then stirred for 1 min and centrifuged (16000× *g*, 0 °C, 5 min). A 400 µL portion of the supernatant was concentrated for 1 h using a centrifugal evaporator (CVE-2000, Eyela, Japan) and freeze-dried overnight. Oxime formation was performed by reacting the freeze-dried sample with pyridine-solubilized methoxyamine hydrochloride (20 mg/mL, 50 µL) at 30 °C for 90 min. Trimethylsilylation was then achieved by treating the sample with MSTFA (100 µL) at 37 °C for 30 min. 

The derivatized samples were analyzed by GC-MS using a GCMS-QP2010 Ultra system (Shimadzu Co., Kyoto, Japan) equipped with an Agilent J&W DB-5 column (length 30 m, internal diameter 0.25 mm, film thickness 1.00 µm, Agilent Technologies, Santa Clara, CA, USA). The GC oven temperature started at 100 °C, held for 4 min, increased to 320 °C at 10 °C/min, and held for 11 min. The injection port temperature was 280 °C. The derivatized sample (1 µL) was injected in split injection mode with a split ratio of 10:1. Helium was employed as the carrier gas at a constant linear velocity of 39.0 cm/s, and the purge flow rate was 5 mL/min. Quadrupoles were used for MS mass separation, and electron impact was used for ionization. The ion source temperature was 200 °C, the interface temperature was 280 °C, and the ionization voltage was 70 eV. Measurements were carried out in scan mode in the range of 45–600 *m*/*z* (unit mass resolution). Retention time correction of peaks (retention index) was carried out based on the retention time of a standard alkane series mixture (C-6 to C-33) using the automatic adjustment of retention time function of the Shimadzu GCMS solution software. Peak annotation was performed using the commercially available GC/MS Metabolite Component Database Ver. 2 (Shimadzu Co., Kyoto, Japan), which contains a mass spectral library. Peaks were annotated under the condition of possessing a similarity index of more than 80 and a target ion with a confirmation ion ratio of ≥50% in absolute tolerance.

### 3.4. Antioxidant Assay

DPPH scavenging activity was measured using the method described by Katsube et al. [42], with modifications. Briefly, a mixture of 30 mg of the powdered sample (from the sample prepared for GC-MS) in 80% ethanol (1 mL) was vortexed, sonicated, and centrifuged (10,000× *g*, 4 °C, 5 min). The supernatant was collected, and the resulting pellet was extracted an additional two times using this procedure. The supernatants were combined and 10 µL of this solution was added to the wells of a 96-well plate and mixed with 200 µL of DPPH working solution (0.4 mM DPPH ethanol solution/400 mM MES buffer solution/ultrapure water). The absorbance of the mixture was recorded at 520 nm within 10 min of mixing using a microplate reader (SpectraMax 340PC384, MOLECULAR DEVICES, Tokyo, Japan). DPPH was expressed as µmol Trolox equivalent (TE)/30 mg sample, using Trolox as the standard.

ORAC was measured using the method described by Prior et al. [43], with modifications. A 150X dilution of the 80% ethanol extract from the DPPH measurements was prepared. The diluted sample solution (25 µL) and 81.6 nM fluorescein (100 µL) were added to the wells of a 96-well plate. After shaking at 1200 rpm for 10 s, the plate was incubated for 30 min at 37 °C. A solution of 2,2′-azobis(2-amidinopropane) dihydrochloride (200 mM, 25 µL) was added to the wells, and the plate was shaken at 1200 rpm for 10 s. The fluorescence intensity (ex/em: 485/528 nm) was then recorded 50 times at 2 min intervals using a microplate fluorometer (Fluoroskan Ascent FL, Thermo Fisher Scientific, Waltham, MA, USA). The area under the curve (AUC) in the spectra was calculated, and the ORAC value was expressed in nmol Trolox equivalent (TE)/30 mg sample, using Trolox as the standard. 

The SPSS STATISTICS 24 software (IBM, Armonk, NY, USA) was used for statistical analysis. One-way ANOVA was used to compare the mean DPPH and ORAC activity values between the cabbage varieties. Significant differences found in antioxidant activity were subsequently tested by Tukey’s multiple comparison test. The significance level was set at 5% (*p* < 0.05).

### 3.5. Electronic Tongue Measurement

The electronic tongue measurements were performed using a previously reported method [39,40], with modifications. Briefly, chopped cabbage stored at −80 °C (50 g) was added to ultrapure water (250 mL) and homogenized with an ACE Homogenizer AM-7 (Nihonseiki Kaisha Ltd., Tokyo, Japan) at 5000 rpm for 5 min over ice. After centrifugation (15,000× *g*, 4 °C, 15 min), 100 mL of the supernatant was collected and diluted to 400 mL. This solution was used to measure the initial taste and aftertaste of the cabbage.

The taste was measured using a TS-5000Z taste sensor system (Insent, Kanagawa, Japan). Each sample solution was tested using six sensor types: AAE (umami), CT0 (saltiness), CA0 (sourness), C00 (acidic bitterness), AE1 (irritant), and GL1 (sweetness). The differences in human perception of taste intensity were estimated using Weber’s law from an average of three repeated measurements, and the resultant value was taken as the intensity of each taste attribute. The system detects two types of tastes, initial taste and aftertaste. In this study, the relative potentials obtained from each sensor probe were used to measure the selected initial tastes. The changes in membrane potential caused by adsorption for C00 (bitterness), AE1 (astringency), and AAE (richness) sensor probes were used to measure the aftertastes [44]. SPSS STATISTICS 24 was used for statistical analysis. One-way ANOVA was used to compare the mean values between cabbage varieties for each taste attribute. Attributes that showed significant differences were subsequently tested by Tukey’s multiple comparison test. The significance level was set at 5% (*p* < 0.05).

### 3.6. Multivariate Analysis

SIMCA 14 (MKS Instruments, Andover, MA, USA) was used for multivariate analysis. The results were evaluated as described by Eriksson et al. [33]. The data sets consisted of the *x* variables (the intensity of each annotated metabolite) and the *y* variables (measured values of each taste attribute and two antioxidant activities). PCA-X or Y, the unsupervised learning analysis without the *y* or *x* variables, respectively, was carried out with unit variance-scaling (UV) to analyze differences in the metabolite components, taste attributes, and antioxidant activity profiles of the cabbage varieties. OPLS-DA was used to clarify the differences between groups of *x* variables. Numbers 1 to 6 were assigned to each variety. The data was normalized with UV, and then the discriminant analysis between cabbage varieties was performed using OPLS-DA. OPLS analysis was used to evaluate the correlation between *x* and *y* variables in order to identify important *x* variables that correlate with each *y* variable by creating a model that predicts *x* from *y*. The model obtained by OPLS analysis and OPLS-DA was considered to have excellent quantitative and predictive performance for R2Y ≥ 0.65 and Q2Y ≥ 0.5 [33]. Model validity and accuracy were assessed using CV-ANOVA and a permutation test. CV-ANOVA is used in OPLS and OPLS-DA significance tests [45]. The significance level was set at 5% (*p* < 0.05). The permutation test was performed with *n* = 200 and evaluated using the R2Y- and Q2-intercept. A corresponding regression equation in OPLS was created for any significant model for a given *y* variable. Additionally, the values of variables important for prediction (VIP) were calculated to identify the characteristic *x* variables that correlate with each *y* variable. The VIP values ≥ 1.0 were considered significant.

## 4. Conclusions

This study correlated the primary metabolites of six cabbage varieties with their characteristic taste and antioxidant activities. Electronic tongue analysis was used to evaluate the nine constituent tastes objectively, while biochemical assays were used to quantitate the antioxidant activities in each variety. As a result, significant predictive models were successfully created for DPPH, sourness, acidic bitterness, irritant, umami, saltiness, bitterness, astringency, and richness. The high-VIP components of each model demarcate characteristic metabolites that show differences between varieties. The metabolites identified in this study as being key factors in cabbage taste and antioxidant activity were 4-aminobutyric acid (DPPH and umami), fructose 1-phosphate (sourness), adipic acid (acidic bitterness), 5-oxoproline (irritant and saltiness), *N*-acetylglycine (bitterness), *O*-phosphoethanolamine (astringency), and homovanillic acid (richness). Additionally, these represent markers indicating breed differences. The success of this study paves the way for future investigations of secondary plant metabolite involvement in cabbage breed-specific characteristics as well as the examination of the effects of growth conditions on metabolite composition and resultant attributes.

## Figures and Tables

**Table 1:** Evaluation of the models obtained from the OPLS analysis of each *y* variable.

y Variable	A ^1^	N ^2^	R2X(cum)	R2Y(cum)	Q2(cum)	*p*-value ^3^	Permutation Test	
							R2Y Intercept	Q2 Intercept
DPPH	1 + 1 + 0	30	0.233	0.866	0.595	0.016	0.668	−0.294
ORAC	1 + 1 + 0	30	0.197	0.770	0.421	0.26	—	—
Sourness	1 + 2 + 0	30	0.319	0.957	0.839	0.000015	0.702	−0.355
Acidic bitterness	1 + 1 + 0	30	0.215	0.874	0.684	0.0010	0.679	−0.314
Irritant	1 + 2 + 0	30	0.308	0.977	0.869	0.0000045	0.685	−0.367
Umami	1 + 2 + 0	30	0.305	0.961	0.858	0.0000046	0.714	−0.294
Saltiness	1 + 2 + 0	30	0.32	0.947	0.792	0.00011	0.729	−0.358
Bitterness	1 + 1 + 0	30	0.242	0.85	0.575	0.022	0.681	−0.321
Astringency	1 + 2 + 0	30	0.287	0.975	0.869	0.000011	0.696	−0.332
Richness	1 + 1 + 0	30	0.259	0.866	0.746	0.000035	0.668	−0.334
Sweetness	1 + 1 + 0	30	0.226	0.835	0.472	0.20	—	—

^1^ A = number of models. ^2^ N = number of samples used in producing the models. ^3^ *p*-values were obtained from the analysis of cross-validated predictive residuals (CV-ANOVA).

## Supplementary Materials

The following are available online. Table S1: List of *x* and *y* variables. Table S2: The *p*-values obtained by Tukey’s multiple comparison test for antioxidant activity (Figure 1) and taste attributes (Figure 2). Table S3: List of VIP values obtained by OPLS analysis. Figure S1: Typical total ion chromatograms obtained by GC-MS analysis of different cabbage varieties. Figure S2: (A) Score plots and (B) loading plots obtained by PCA-X analysis of cabbage varieties. Figure S3: (A) Score plots and (B) loading plots of OPLS-DA results for the different cabbage varieties.
