# Supplementary material for: Analysis of Primary Metabolites in Cabbage (Brassica oleracea var. capitata) Varieties Correlated with Antioxidant Activity and Taste Attributes by Metabolic Profiling
Source: Molecules. 2019 Nov 25;24(23):4282. doi: 10.3390/molecules24234282 (PMC6930592; doi:10.3390/molecules24234282)
Supplement: Supplementary file 1 [file molecules-24-04282-s001.zip › Fig. S.pdf]

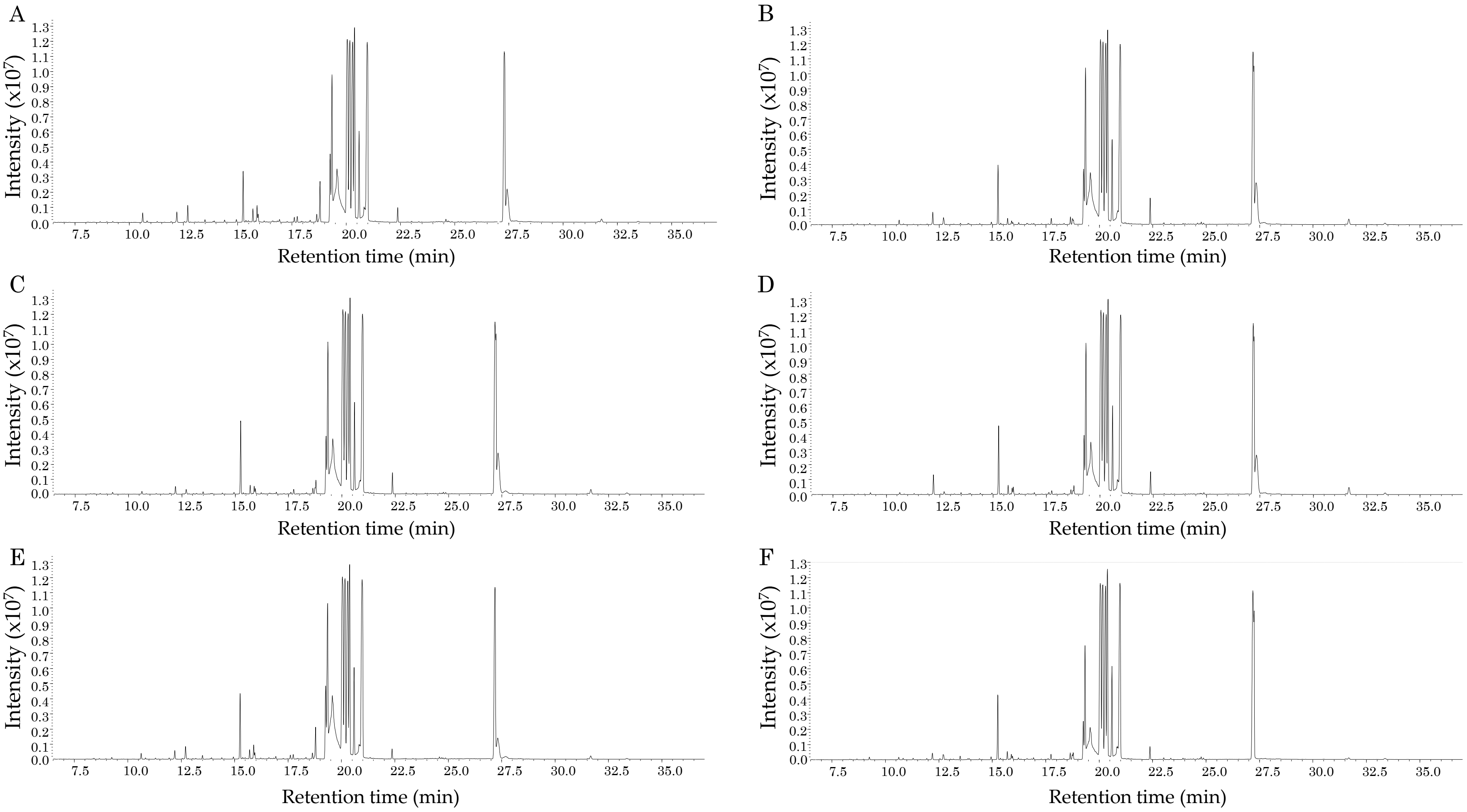

Figure S1 Typical total ion chromatograms obtained by GC-MS analysis of different cabbage varieties. (A) YR-GINJIRO, (B) SATOU-KUN, (C) OKINA, (D) KINKEI-201GO, (E) KOGETSU-SP, (F) HIRO-KANRANS.

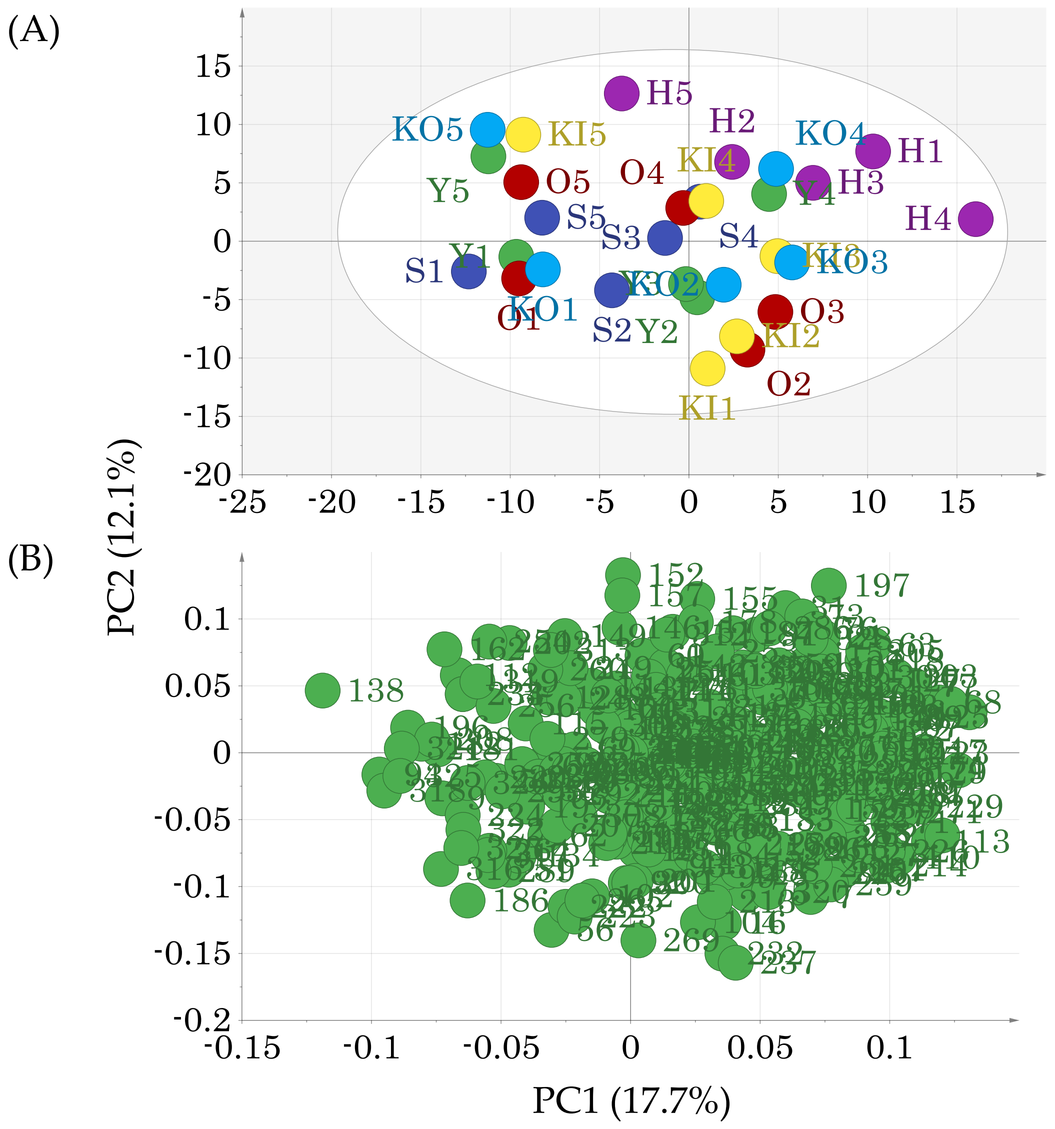

Figure S2 (A) Score plots and (B) loading plots obtained by PCA-X analysis of cabbage varieties. Letters and numbers in (A) indicate sample ID and numbers in (B) indicate x variables ID (Table S1).

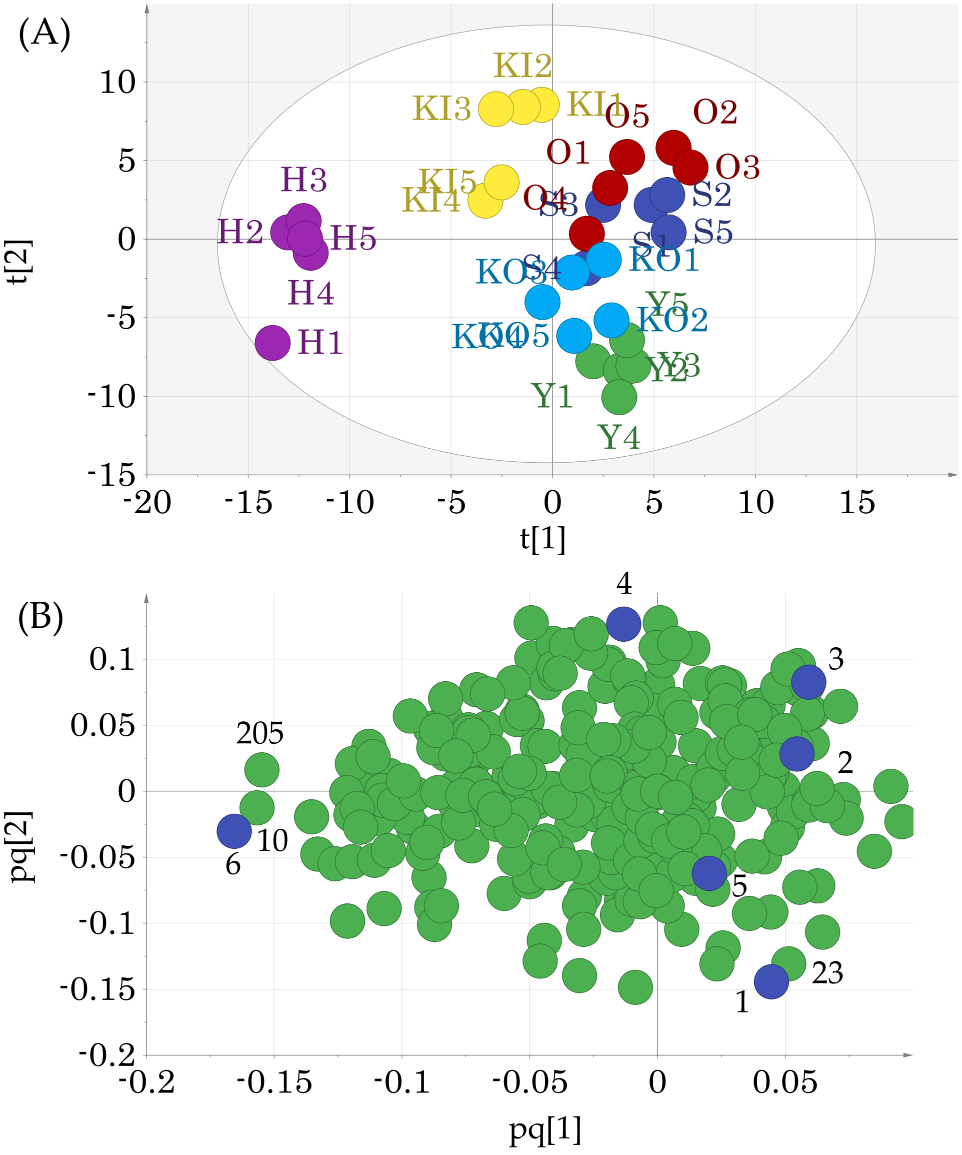

Figure S3 (A) Score plots and (B) loading plots of OPLS-DA results for the different cabbage varieties. Letters and numbers in (A) indicate sample ID and numbers in (B) indicate group identity (blue circles) (Table S1). Numbers adjacent to green circles are x variable IDs in Table S1.
